# Heat Wave Vulnerability Mapping for India

**DOI:** 10.3390/ijerph14040357

**Published:** 2017-03-30

**Authors:** Gulrez Azhar, Shubhayu Saha, Partha Ganguly, Dileep Mavalankar, Jaime Madrigano

**Affiliations:** 1 The RAND Corporation, 1776 Main Street, Santa Monica, CA 90401, USA; jmadriga@rand.org; 2 Pardee RAND Graduate School, 1776 Main Street, Santa Monica, CA 90401, USA; 3 Rollins School of Public Health, Emory University, Atlanta, GA 30322, USA; shubhayu@gmail.com; 4 Indian Institute of Public Health, Gandhinagar, Gujarat 382042, India; psganguly@iiphg.org (P.G.); dmavalankar@iiphg.org (D.M.); 5 Public Health Foundation of India, New Delhi 110070, India

**Keywords:** heatwave, vulnerability, heat vulnerability index, vulnerability assessment, mapping, India

## Abstract

Assessing geographic variability in heat wave vulnerability forms the basis for planning appropriate targeted adaptation strategies. Given several recent deadly heatwaves in India, heat is increasingly being recognized as a public health problem. However, to date there has not been a country-wide assessment of heat vulnerability in India. We evaluated demographic, socioeconomic, and environmental vulnerability factors and combined district level data from several sources including the most recent census, health reports, and satellite remote sensing data. We then applied principal component analysis (PCA) on 17 normalized variables for each of the 640 districts to create a composite Heat Vulnerability Index (HVI) for India. Of the total 640 districts, our analysis identified 10 and 97 districts in the very high and high risk categories (> 2SD and 2-1SD HVI) respectively. Mapping showed that the districts with higher heat vulnerability are located in the central parts of the country. On examination, these are less urbanized and have low rates of literacy, access to water and sanitation, and presence of household amenities. Therefore, we concluded that creating and mapping a heat vulnerability index is a useful first step in protecting the public from the health burden of heat. Future work should incorporate heat exposure and health outcome data to validate the index, as well as examine sub-district levels of vulnerability.

## 1. Introduction

The Intergovernmental Panel on Climate Change (IPCC) report [1] highlights the projected increases in heatwave frequency, intensity and duration, and resulting deaths both globally and in India. Heatwave events have caused massive deaths in the past; the most famous among them are the European 2003 and Russian 2010 heatwaves, where tens of thousands died [2,3,4]. India has experienced several heatwaves, and most recently, just in the past two years, thousands have reportedly died [5]. Research has documented an increase in cardiovascular [6], respiratory [7], and all-cause [6] mortality along with increases in ambulance calls and admissions [8,9] resulting from heatwave exposure. While most of the evidence is from North America and Europe, there is an emerging body of evidence from developing countries, including India [10], where heat wave deaths may currently be underestimated [11].

At the same time, heat-related deaths are preventable and prevention programs have been shown to be extremely cost effective [12]. Population adaptation [13] along with preparedness measures have reduced mortality. Indeed, several cities and countries around the world have adopted heatwave preparedness plans [13]. However, in India, this effort is limited to only a few cities [14]. A broader preparedness strategy is particularly important given the large population, difficult local conditions, and limited adaptive capacity.

Health vulnerability can be conceptualized as complex and multidimensional [15]. Vulnerability encompasses individual biophysical characteristics, as well as population-level socio-economic-environmental characteristics. These population measures have typically included measures of age, income, discrimination, social isolation, vegetation, and health characteristics [16]. Incorporating multidimensional data can present a more comprehensive characterization of vulnerability. Given the considerable intra-country variations in these measures that exist, it is prudent to use this characterized vulnerability to identify communities in need of prioritized and focused interventions. Heat Vulnerability Indices (HVI) have been found to be a useful screening tool for targeting heat risk interventions [16].

Several international studies have explored vulnerabilities at the national, county, and city levels [17,18,19,20], but none have comprehensively examined India. Additionally, most of this work has been performed in the context of urban settings, while the majority of the Indian population resides in rural areas. To our knowledge only one Indian study looked at agricultural vulnerability using census data from 2001 [21]. In fact, a recent review of heat vulnerability indices points out that that the majority of studies have been performed in Europe and the United States and recommends further study in other countries and regions to account for local context [16]. We, therefore, aim to create and map an integrated district level heat vulnerability index for India that can be used to identify the most heat-vulnerable districts in the country.

## 2. Materials and Methods

### 2.1. Data Sources

Data was extracted from the Census of India 2011, District Level Household Survey (DLHS)-3 data [22], and from the Indian Space Research Organization ISRO server Bhuvan. District level census data was downloaded through the census of India portal. We chose districts as the unit for our analysis. While states are too heterogenous, districts are appropriate for planning purposes, and are the smallest unit for which we could get reliable data from multiple sources. We downloaded data for household amenities and the primary census abstract (PCA). Some DLHS variables were extracted from the Annual health survey report from the ministry of health and family welfare website. Satellite data was extracted from the ISRO server’s “Bhuvan” tool where the average vegetation fraction and Normalized Difference Vegetation Index (NDVI) images were layered with a district level India shapefile and these variables (mean, median, maximum, minimum, range, and standard deviation) were calculated for each of the district polygons using GIS software.

### 2.2. Choice of Variables

Our initial dataset had 140 variables for 640 districts. Based on the existing literature [17] and removal of duplicative variables that represented similar constructs, we shortlisted variables across the demographic, social, economic, health, and environmental domains. Researchers independently considered variables for inclusion based on knowledge from prior literature. Final variables were then compared and conflicts were resolved through discussion. We were left with 17 variables to be entered for PCA analysis. We did not assign weights to individual variables.

The shortlisted variables plausibly (and have been documented to) affect the heat-health relationship. The final list of variables employed in analysis along with their respective sources are listed in Table 1.

Demographic variables included those based on age and gender. For age extremes, we used the percentage of elderly and under five and for gender we used the sex ratio. While studies have documented elderly age to be a risk factor [3,9,23,24,25,26,27,28,29,30,31,32], there is an argument that children [32,33] could also be at a higher risk, given their higher metabolic rate.

Social class was represented through the percentage of people belonging to the scheduled castes (Dalits) and scheduled tribes (Adivasis). These groups are recognized by the Indian constitution as depressed classes and are a target for development and affirmative action programs. We used these as substitutes for race in the Indian context. Though there are no studies which highlight their vulnerability to extreme heat, this is perhaps from an absence of such literature. Studies between ethnic groups and heat-related deaths show equivocal effects [27,34,35]. This may be due to differential ownership of household amenities, chiefly air conditioning.

Socio economic variables were those of literacy, defined as an ability to read and write in any language; and occupational status of a worker, defined as producing goods and services. Education has been seen to be associated with heat mortality [6,30] perhaps from increased awareness and also as a proxy for socio economic status. Similarly, being in a worker status would increase the environmental exposure to heat since the majority of workers work in the agricultural sector as cultivators and agricultural laborers. Income was assessed through the percentage of people in the lowest wealth quintile. Several studies including those from Asia and India have documented the effects of poverty on heat-related deaths [6,25,29,32].

Household amenities were assessed through the presence of household amenities such as the presence of drinking water inside premises, living in a good house, having a mobile phone, radio and TV [32]. While much research has gone into analyzing the effects of air-conditioning [34,36], we did not include this variable because its prevalence is quite low, electricity supply is irregular across the country, and approximately 300 million people have no power connection. However, many of the household amenities assessed require electricity and can serve as a proxy for electricity supply.

Land cover was assessed through Vegetation Fraction (VF) and Normalized Difference Vegetation Index (NDVI). Studies have documented the protective influence of green cover from heat island effects and heat deaths [37] and these have been included in other indices [17].

Population health was assessed through the immunization status children (12–23 months) fully immunized (BCG, 3 doses each of DPT, and Polio and Measles) (%) and presence of a health facility (sub-center) within 3 km. Preexisting illnesses have been documented to affect heat mortalities [32]. Because we were unable to obtain prevalence of chronic disease that may be most closely linked to heat vulnerability (e.g., diabetes, cardiovascular diseases) at the district level, we used these factors as a proxy for overall underlying population health status.

### 2.3. Data Analysis

The data were merged using the census district numbers as unique identifiers. They were then manually checked for any discrepancies such as those between district names and were resolved through crosschecking. Where district level data were missing, we substituted state-wide averages for the districts.

Data were analyzed with STATA ver. 13, 2016 (StataCorp LP, College Station, TX, USA) and qGIS 2.10.1 (Open Source Geospatial Foundation Project). The map was created with R v. 3.3.2 (R Foundation for Statistical Computing, Vienna, Austria). All data used in this study were publicly available data and did not contain any individually identifiable information.

We calculated Spearman’s correlation coefficients between the vulnerability variables. We then employed the PCA technique to reduce the dimensionality of these variables. In order to perform PCA variables need to be on the same scale, we normalized all our variables by calculating their *Z* scores. All variables were in the same direction i.e., increasing value implies an increasing vulnerability. After application of PCA we tested for unexplained variation and adequacy of analysis by Kaiser-Meyer-Olkin (KMO) test. We retained four factors based on these criteria—Kaiser’s criteria of eigenvalues > 1, break in values in the scree plot test, and the variance explained by the factors. We tried factor rotations to increase the variability among factors. Individual factor scores were predicted for each district. In absence of any information with regards to the shape or interaction of factors, we assumed a linear relationship and calculated the heat vulnerability index by summing up these district level factor scores.

## 3. Results

Table 1 demonstrates the sources and descriptive characteristics of the 17 raw variables included. Some of these variables showed interesting variations, for example, on average only 42.3% of households had drinking water inside their premises but it ranged from 2.4% to 93.8% across districts. Similarly TV ownership, immunization status, having nearby health sub-centers and vegetation also showed such marked variations.

Variable correlations showed many of the variables highly correlated with each other at the 0.001 significant level. (not shown here).

Table 2 shows the PCA results with Varimax rotation. The factors have been reduced to four dimensions. These correspond to demographic, socio-economic, vegetation, and health systems. The PCA led to four factors with primary loadings, these appeared to be (1) demographic; (2) socio-economic; (3) environmental; and (4) health factors. Demographic loadings were constituted of extremes of age, socio-economic loadings were driven by household amenities, environmental loadings were contributed by the VF and NDVI scores and health was driven by availability of health facilities nearby.

**Figure 1:**
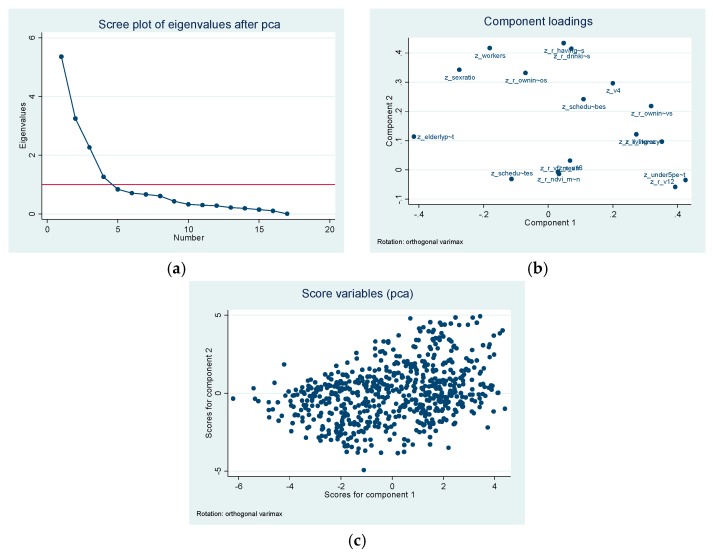
PCA results. (**a**) Scree plot of Eigenvalues after PCA; (**b**) component loadings on orthogonal (Varimax) rotation; (**c**) score variables on orthogonal (Varimax) rotation.

Of the 17 variables that we started with, these four groups of factors were able to account for 78% of the total variation. The scree plot of eigenvalues showed a clean break at four components (Figure 1). This was also in agreement of the Kaiser criteria. The Kaiser–Meyer–Olkin (KMO) test shows adequacy of our analysis (>0.50).

**Figure 2:**
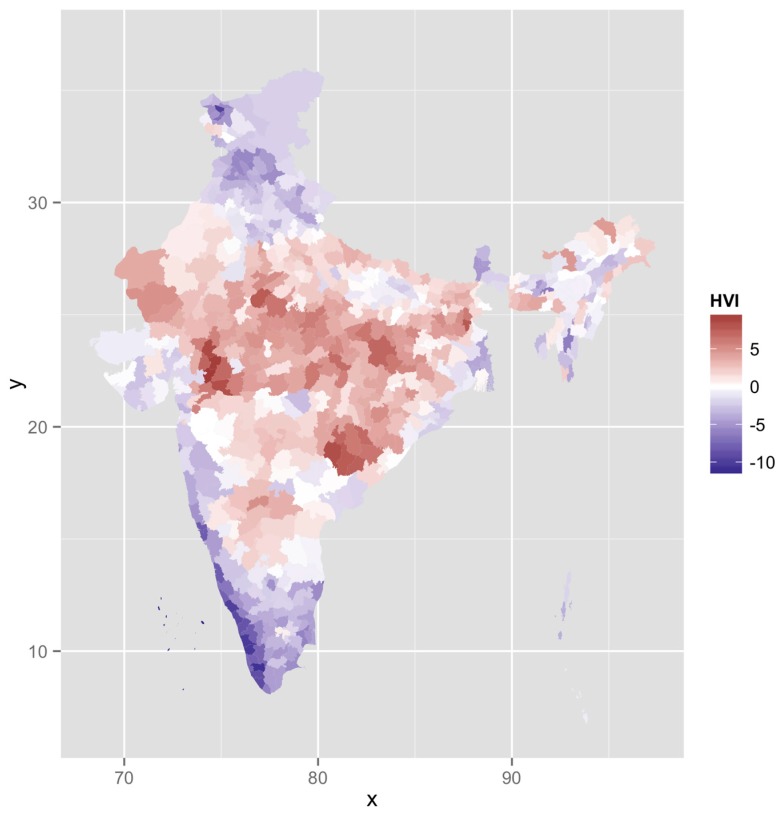
HVI mapping.

The HVI calculated as a sum of the four individual factors for each district ranged from −11.8 to 9.4, it had a mean close to 0 and SD of 3.5. Figure 2 maps the vulnerability across the country. Spatial clustering of these “hot spots” is observed in central India. These districts have poor socio-economic and development indicators and appear to be high on the heat vulnerability index.

We classified these categories based on the SD scores as “very high” (>2SD), “high” (1–2SD), “high normal” (0–1SD), “low normal” (−1–0SD), “low” (−2–1SD), and “very low” (<−2SD). We chose this SD based classification instead of equal categorization to better represent the variation. Table 3 shows the number of districts according to HVI standard deviations.

Ten districts had an HVI score of “very high” (>2SD), most of them in central India in the states of Madhya Pradesh and Chhattisgarh (Table 4). Twenty districts had an HVI score of “very low”; most of them were in the relatively developed states of Kerala and Goa and union territories of Lakshadweep.

## 4. Discussion

This study provides a relative ranking of heat wave vulnerability for all districts in India. Although much is known about factors that contribute to vulnerability from other settings [16,17,18,19], there has been minimal research conducted within India on heat-related vulnerability. By coupling this knowledge with local context and using methods previously applied in other settings [17], we created an index that describes relative variation in heat-related vulnerability across all of India. This index can be used by planners, policy makers, and disaster mitigation experts to target climate adaptation efforts.

Similar to the findings of other international studies [17], our index too identified demographic, socio-economic, environmental, and health system factors. However, there are important differences in the choice of initial variables making this index useful to the Indian and developing country settings.

The high and very high HVI districts were in the central part of the country. With a higher tribal population, these states have been at the lower end of various health, education, economic and population growth indicators. They are referred to as the Empowered Action Group (EAG) states and often targeted for focused interventions. These land-locked, high HVI districts in the North and Central Indian plains are classically known as the “heat belt”.

While the use of air conditioning has been observed to have the greatest impact in reducing heat wave deaths in the US [36] it is unlikely to be a solution for India at least in the short term because of lack of a reliable and continuous power supply, the high cost and low penetration of air conditioning.

Suitable local adaptation strategies therefore need to be considered. These may include a range of measures, some of which have been discussed in the literature, such as public messaging (Radio, TV), mobile phone-based text messages, automated phone calls, and amber alerts; to others such as traditional adaptation practices of staying indoors, wearing comfortable clothes, and diets. These are often visible in terms of the housing design and construction material used. Simple design features such as having shaded windows and underground water storage tanks can be helpful. Use of insulator housing materials similarly can be an effective method of prevention. Having access to drinking water within housing premises and indoor toilets could be important. We chose several household amenities not just to proxy for income but also for their protective role.

For risk management, it would make sense to observe whether these identified areas of high vulnerability are also the same as those with higher temperatures and humidity. Similarly, had district level heat wave death data been available, we could have used it to validate this index. Our index does show high (>0.70) and significant correlations with literacy rates, low income status, TV ownership, having toilets and drinking water and open defecation practices. These could be seen as starting points framing local adaptation strategies. These correlations highlight the importance of interventions against other associated diseases such as gastrointestinal diseases in children and water-borne illnesses etc. There is a moderate correlation of 0.42 between HVI and average summer land surface temperatures (from satellite data) suggesting a relationship between higher temperatures and heat vulnerability. The index also shows moderate negative correlation (–0.46, *p* < 0.001) with urbanization signifying a possible greater vulnerability threat in rural areas. Since the majority of Indians reside in rural areas, this could have important implications. Outdoor workers have been identified as being at a greater risk during heatwaves. In rural settings, agricultural practices in different regions of India may also have diverse vulnerability patterns.

Some limitations of this study also arise from availability of data. Cardiovascular and/or respiratory diseases are more closely related to heat vulnerability but prevalence of chronic diseases at the district level was not available. Similarly, there was no pan India district level data on social isolation or electricity. For the three DLHS variables, we had missing data for the state of Nagaland. We used state averages instead. However, since Nagaland is a small state which has not reported heatwave deaths, we believe this substitution is unlikely to have major effects. In calculating HVI, we assumed a linear combination of factors with no weighting as a good first assumption. Inclusion of temperature as an exposure variable could have been helpful but because temperatures vary at country-wide levels in a thermally diverse India, it would serve to bias the index in favor of places with higher normal temperatures. Our approach is in line with established methods [17] for large areas. Also, district level temperature data is only collected for a small fraction of the total 640 districts. In view of still building evidence base from Indian temperature-mortality studies we have cited western literature and some Asian and Indian studies identifying vulnerability factors. This approach has also been demonstrated previously in the air pollution literature [38].

In many prior heat vulnerability studies, rural areas have been overlooked, but may have high vulnerability, and this may be especially important in India given the importance of poverty, and agricultural livelihoods in mediating the temperature mortality relationship. However, since the district level data includes both rural and urban areas; by aggregating them we may have missed the differences between these patterns of vulnerability [39,40]. Also, since we only had district level data, if such data was available at the finer block (Taluka) level, we would have a better identification of vulnerable areas. Similarly, intra-city vulnerability patterns would have been interesting to observe given availability of urban ward level data. Given data availability, future work could also identify areas using the Koeppen climate classification assuming that the warm, dry, arid, and humid areas are more vulnerable. As research continues, we may identify more complex relationships and therefore we could conceive of other heat vulnerability indices with non-linear relationships and differential weights.

Despite the above discussed limitations there are several strengths to this work. This is the first study to look at heat vulnerability across India. It provides a preliminary screening to target heat-health and climate adaptation efforts. This methodology can be used to for further investigations into vulnerability.

## 5. Conclusions

We developed a heat wave vulnerability index that aggregates indicators across several dimensions for all districts in India. This index can be used in initial efforts to target resources for adaptation efforts. Most heat preparedness plans are designed and implemented at a local level, and this index can help identify metropolitan areas that are at highest risk. Since heat wave vulnerability varies across spatial scales, our methods can be extended for sub-district level analysis and modified to develop urban and rural indices. Further work within this context should include testing the sensitivity of our linear combination assumption and validating this index to health outcomes.

## Figures and Tables

**Table 1:** Heat-health vulnerability data for 640 districts of India.

	Category	Source	Variable	Mean	Standard Deviation	Minimum	Maximum
1	Demographic	Census 2011	Elderly (%)	6.941611	1.85144	1.911948	16.31032
2	Demographic	Census 2011	Under five (%)	11.77559	2.430698	6.39814	19.94178
3	Demographic	Census 2011	Sex ratio	945.4773	60.60111	533.5676	1184.402
4	Social Class	Census 2011	Scheduled castes (%)	14.85952	9.127914	0	50.17002
5	Social Class	Census 2011	Scheduled tribes (%)	17.70213	26.97455	0	98.57509
6	Socio-economic	Census 2011	Literacy (%)	62.4771	10.52398	28.77288	88.73746
7	Socio-economic	Census 2011	Workers (%)	41.19976	7.02642	25.83138	66.8953
8	Socio-economic	DLHS 3	Lowest wealth quintile (%)	18.69547	17.8634	0	85
9	Household Amenities	Census 2011	Drinking water inside premises (%)	42.35347	22.93822	2.426598	93.86555
10	Household Amenities	Census 2011	Living in a good house (%)	51.01322	14.27142	13.01783	88.05314
11	Household Amenities	Census 2011	Having only mobiles (%)	51.21369	14.46154	7.97389	79.62046
12	Household Amenities	Census 2011	Owning radios (%)	20.44393	11.38917	2.827992	77.2401
13	Household Amenities	Census 2011	Owning TVs (%)	43.6372	24.04314	5.787766	95.40281
14	Population Health	DLHS 3	Children (12–23 months) fully immunized (%)	56.89797	21.96324	3.8	100
15	Population Health	DLHS 3	Villages having Sub-Center within 3 km (%)	69.91922	18.23694	0	100
16	Land Cover	ISRO	Vegetation Fraction	73.24128	38.98999	10.60944	255
17	Land Cover	ISRO	Normalized Difference Vegetation Index	84.19634	32.20057	35.78857	255

**Table 2:** Factor loadings from varimax rotation based on data from 640 districts.

Variable	Factor 1	Factor 2	Factor 3	Factor 4
Elderly	–0.41	0.11	0.13	–0.19
Under five	0.42	–0.03	–0.01	–0.05
Sex ratio	–0.27	0.34	–0.10	–0.25
Scheduled castes	–0.11	–0.03	0.38	–0.20
Scheduled tribes	0.11	0.24	–0.30	0.24
Literacy	0.35	0.10	0.10	–0.04
Workers	–0.18	0.42	–0.01	0.33
Lowest wealth quintile	0.20	0.30	0.13	–0.18
Drinking water inside premises	0.07	0.41	0.01	0.07
Living in a good house	0.27	0.12	0.02	–0.39
Having only mobiles	0.05	0.43	–0.10	–0.07
Owning radios	–0.07	0.33	0.30	0.09
Owning TVs	0.32	0.22	0.03	–0.15
Children (12–23 months) fully immunized	0.39	–0.06	0.02	0.10
Villages having sub-center within 3 km	0.07	0.03	0.08	0.66
Vegetation fraction	0.03	–0.01	0.55	0.10
Normalized difference vegetation index	0.03	–0.01	0.55	0.08

**Table 3:** Number (%) of districts by HVI (Heat Vulnerability Index) Categories.

HVI Category	Number (%) of Districts
Very high	10 (1.56)
High	97 (15.16)
High normal	213 (33.28)
Low normal	225 (35.16)
Low	75 (11.72)
Very low	20 (3.13)

**Table 4:** Districts with a “very high” HVI score.

District	State
Dakshin Bastar Dantewada	Chhattisgarh
Pakur	Jharkhand
Alirajpur	Madhya Pradesh
Sheopur	Madhya Pradesh
Barwani	Madhya Pradesh
Banswara	Rajasthan
Jhabua	Madhya Pradesh
Malkangiri	Odisha
Dohad	Gujarat
Bijapur	Chhattisgarh
